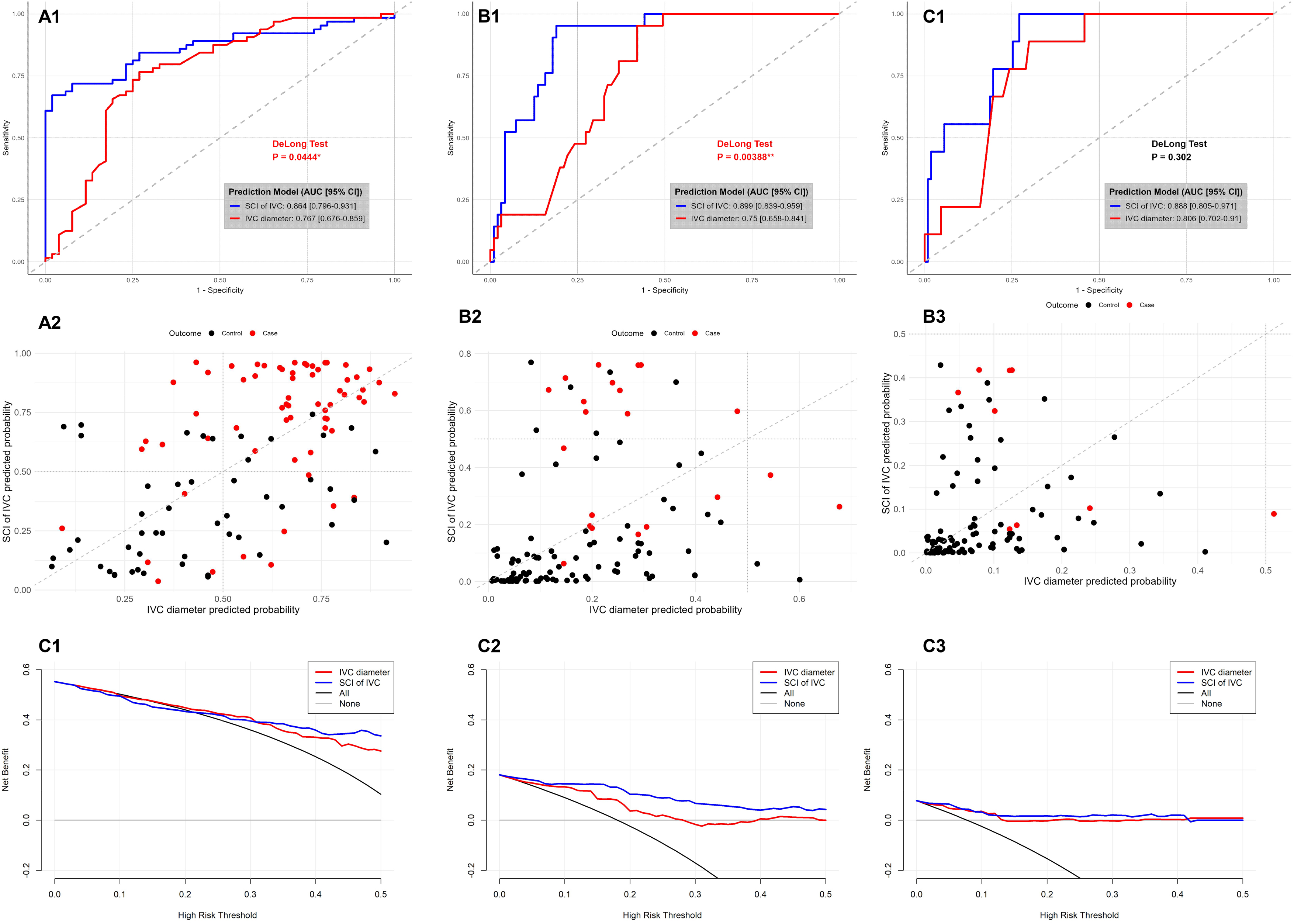# Assessing venous congestion in critical illness: advantages of the inferior vena cava shape change index over diameter

**DOI:** 10.1016/j.aicoj.2026.100032

**Published:** 2026-02-09

**Authors:** Lizhi Li, Yuehua Xu, Xiukai Chen, Wei Huang

**Affiliations:** a Department of Critical Care Medicine, The First Affiliated Hospital of Xiamen University, School of Medicine, Xiamen University, Xiamen, China; b School of Clinical Medicine, Fujian Medical University, Fuzhou, Fujian, China; c Department of Critical Care Medicine, Zhao'an County Hospital, Zhangzhou, China

**Keywords:** Venous excess ultrasound, Shape change index, Inferior vena cava, Venous congestion

## Abstract

**Background:**

The traditional Venous Excess Ultrasound (VExUS) scoring system relies on inferior vena cava (IVC) diameter measurements, which are affected by multiple confounding factors. The Shape Change Index (SCI) of IVC, defined as the ratio of short-axis diameter to long-axis diameter, may provide a more stable morphological indicator of venous filling.

**Methods:**

In this prospective study, trained operators performed bedside ultrasonography to measure IVC diameter and calculate the SCI of IVC (short-axis diameter/long-axis diameter). Hepatic, portal, and renal venous Doppler waveforms were used to grade venous congestion (Grade 0–3). Diagnostic performance of SCI and IVC diameter for detecting venous congestion was evaluated using receiver operating characteristic(ROC) analysis, with additional exploratory analyses performed to further characterize incremental diagnostic value.

**Results:**

A total of 116 venous Doppler examinations from 84 critically ill adults were analyzed. The SCI of IVC demonstrated a stronger correlation with venous congestion grade than IVC diameter (Spearman’s ρ = 0.691 vs 0.490, both p < 0.001). For detecting any venous congestion (VExUS Grade ≥1 vs Grade 0), the SCI of IVC showed significantly better diagnostic discrimination than IVC diameter, with an area under the curve of 0.864 compared with 0.767, respectively (p = 0.044). The diagnostic advantage of SCI appeared to be more evident in examinations without advanced venous congestion. Higher SCI values were associated with greater disease severity.

**Conclusions:**

The SCI of IVC demonstrates superior diagnostic performance compared with IVC diameter for the identification of venous congestion in critically ill patients, particularly at earlier or less advanced stages. These findings support the use of SCI as a complementary screening tool within the existing VExUS framework. Further multicenter studies are required to confirm its clinical utility and generalizability.

## Background

In the hemodynamic management of critically ill patients, clinical focus has shifted from evaluating fluid responsiveness—whether fluid administration increases cardiac output—to assessing fluid tolerance, which determines whether the patient can receive fluids without developing organ congestion [[1], [2], [3]]. Fluid responsiveness and fluid intolerance can coexist in critically ill patients, emphasizing the need for accurate tools to assess venous congestion [4]. To address this, the Venous Excess Ultrasound (VExUS) scoring system has emerged as a novel bedside ultrasound tool for comprehensive, noninvasive assessment of systemic venous congestion [5]. By integrating inferior vena cava (IVC) diameter measurements with Doppler waveform analyses of the hepatic, portal, and renal veins, VExUS provides a unified scoring framework that reflects overall venous hemodynamics [5,6].

VExUS encapsulates the dynamic interaction between venous return and cardiac function, rather than serving as a simple indicator of volume overload [7]. Previous studies have shown that VExUS reflects venous congestion within the framework of venous return physiology, providing dynamic information on the pressure gradient between mean systemic filling pressure and right atrial pressure, rather than relying on right atrial pressure alone [8]. Elevated VExUS scores have been associated with higher risks of acute kidney injury (AKI), organ dysfunction, and adverse clinical outcomes in both cardiac and general ICU settings [5,[8], [9], [10], [11]]. VExUS-guided therapy allows more precise titration of fluid administration or removal, helping clinicians avoid both fluid overload and hypovolemia [9,[11], [12], [13], [14]]. Collectively, this evidence positions VExUS as a promising, noninvasive bedside tool for optimizing hemodynamic management in critically ill patients.

However, the conventional VExUS framework relies primarily on the absolute IVC diameter (≥2 cm) to identify venous congestion. Increasing evidence suggests that the Shape Change Index (SCI) of IVC—defined as the ratio of short-axis to long-axis diameters—offers a more stable and physiologically relevant reflection of intravascular pressure changes than absolute diameter alone [15]. The rationale lies in the IVC’s dynamic morphological response to pressure fluctuations: under hypovolemic conditions, the vessel assumes an elliptical shape due to reduced transmural pressure, while increasing venous pressure has been shown to induce progressive circularization of the IVC cross-section in mechanically ventilated ICU patients [15], a phenomenon that is physiologically consistent with venous distension under elevated transmural pressure. Thus, SCI may serve as a more sensitive indicator of venous distension, better capturing transmural pressure and vascular compliance than static diameter measurements.

Furthermore, SCI may reduce measurement variability caused by anatomical differences, inconsistent probe positioning, and irregular IVC morphology under low central venous pressure(CVP) [15,16]. This makes it a potentially more reliable marker for evaluating venous congestion.

Based on this rationale, the present study aimed to investigate the diagnostic value of the SCI of IVC in assessing venous congestion in critically ill patients. We hypothesized that, compared with traditional IVC diameter, SCI demonstrates a stronger correlation with the severity of venous congestion as defined by VExUS grading and provides superior diagnostic accuracy for its detection.

## Methods

### Study design and population

This prospective, single-center observational study was conducted in the Intensive Care Unit (ICU) of the First Affiliated Hospital of Xiamen University between May and October 2025. Consecutive adult patients admitted to the ICU were screened for eligibility. The ultrasound/VExUS examinations were mainly performed within 24 h of ICU admission. Repeated measurements were taken at variable intervals according to clinical need.

Patients admitted to the intensive care unit during the study period were screened for eligibility. Inclusion criteria were adult patients (≥18 years) who underwent bedside ultrasound evaluation with VExUS assessment for clinical indications related to hemodynamic or fluid status assessment. Exclusion criteria were predefined and included age <18 years, length of ICU stay <24 h, ongoing extracorporeal membrane oxygenation support, end-stage renal disease with renal atrophy, end-stage liver cirrhosis, recent abdominal surgery, and documented Do-Not-Resuscitate status. Patients in whom VExUS or IVC assessment could not be adequately performed or interpreted due to poor ultrasound image quality were also excluded. Eligible patients who met all inclusion criteria and had adequate ultrasound image quality were included in the final study cohort.

The study protocol was approved by the institutional ethics committee (Approval No. 098, 2025). The requirement for informed consent was waived, as all ultrasonographic data were obtained during routine clinical care and anonymized before analysis.

### Data collection and ultrasound protocol

Baseline demographic and clinical data, including the Acute Physiology and Chronic Health Evaluation II (APACHE II) and Sequential Organ Failure Assessment (SOFA) scores, were recorded at ICU admission. All bedside ultrasound examinations were performed by trained operators using a standardized ultrasound system (Navi S, Shenzhen Wisonic Medical Technology Co., Ltd., China). For analytical purposes, all analyses were conducted in a cross-sectional manner, with each ultrasound examination treated as an independent observation reflecting the contemporaneous venous congestion status; no longitudinal or time-dependent analyses were performed.

### Operator training and quality assurance

Before the study, all operators completed a structured training program comprising a four-hour instructional video series on the VExUS protocol (developed by the Beaubien-Souligny group [5]) and a hands-on session supervised by an ICU attending physician experienced in point-of-care ultrasonography. Competency was verified through a practical certification examination. For quality control, ambiguous findings were independently reviewed by two operators, and discrepancies were resolved by consensus.

### Measurement procedure and venous congestion grading

**Fig. 1:**
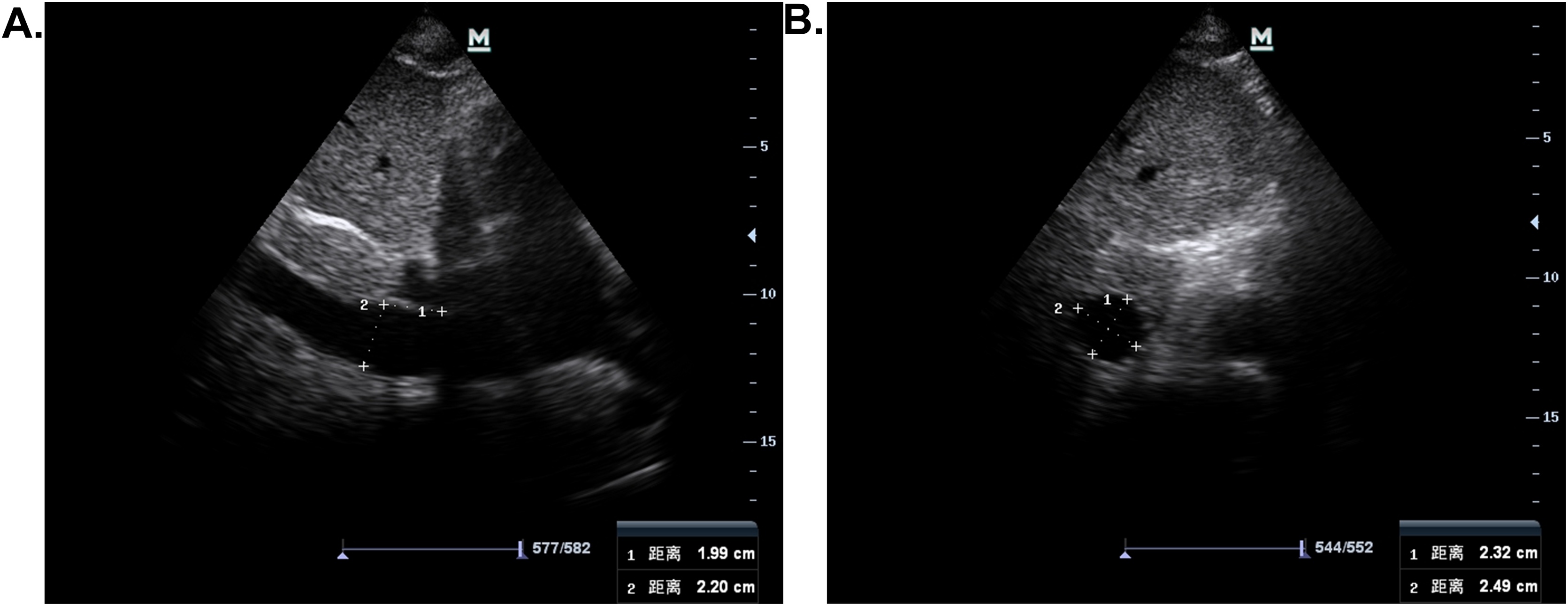
Measurement of the long and short axes of the inferior vena cava. (A) The IVC was visualized in the longitudinal plane via the subxiphoid approach. The measurement location is illustrated at approximately 2 cm from the IVC–right atrial junction along the IVC. (B) The probe was then rotated 90 ° clockwise to acquire a transverse view, ensuring the abdominal aorta, when visible, appeared circular, confirming orthogonal alignment.

Each ultrasound examination included IVC assessment and Doppler evaluation of the hepatic, portal, and intrarenal veins.The IVC was initially visualized in the longitudinal plane via the subxiphoid approach. Measurements were obtained approximately 2–3 cm from the IVC–right atrial junction along the IVC. The probe was then rotated 90 ° clockwise to obtain a transverse view, ensuring the abdominal aorta, when visible, appeared circular, confirming orthogonal alignment (Fig. 1)[15]. Images were frozen at end-expiration, and both the long-axis (L) and short-axis (D) diameters were measured. The SCI of IVC was calculated as the ratio of D to L (SCI = D / L). Two measurements were taken for each parameter, and their mean values were used for analysis.

Hepatic vein Doppler flow was classified as mildly abnormal when the systolic (S) component was reduced but remained hepatopetal, and as severely abnormal when the S wave reversed direction (hepatofugal) [5].

Portal vein Doppler was deemed mildly abnormal when phasic variability (pulsatility) was between 30% and <50%, and severely abnormal when ≥50% [5].

Intrarenal venous Doppler was categorized as mildly abnormal when both systolic (S) and diastolic (D) phases were present but discontinuous, and as severely abnormal when only a monophasic diastolic component was observed [5].

Venous congestion was graded independently of IVC diameter, based solely on Doppler flow abnormalities as follows:

Grade 0 (No congestion): Normal flow in all veins.

Grade 1 (Mild congestion): Mild abnormalities without any severe pattern.

Grade 2 (Moderate congestion): Severe abnormality in at least one Doppler pattern.

Grade 3 (Severe congestion): Severe abnormalities in multiple Doppler patterns.

All echocardiographic measurements were performed by two experienced intensivists and archived for blinded analysis.

### Statistical analysis

Continuous variables were expressed as mean ± standard deviation (SD) or median (interquartile range, IQR), as appropriate. Categorical variables were summarized as counts and percentages. The relationship between venous congestion grades and IVC diameter categories (<2 cm vs. ≥2 cm) was illustrated using stacked bar charts. Associations between congestion grade (0–3) and continuous ultrasound metrics (IVC diameter and SCI) were analyzed using Spearman’s correlation coefficient (ρ) and simple linear regression (R²). Diagnostic performance for detecting venous congestion was primarily evaluated using a unified endpoint (Grade ≥1 vs Grade 0). Severity-stratified ROC analyses (Grade ≥2 and ≥3) were performed as exploratory analyses and are reported in the Supplementary Materials. As exploratory analyses, the incremental predictive value of SCI over IVC diameter was quantified using the Net Reclassification Improvement (NRI) and Integrated Discrimination Improvement (IDI) indices. Clinical utility was further assessed using Decision Curve Analysis (DCA). All analyses were performed using R software (version 4.4.2), and a two-sided p value <0.05 was considered statistically significant.

## Results

### Baseline characteristics and clinical outcomes

**Fig. 2:**
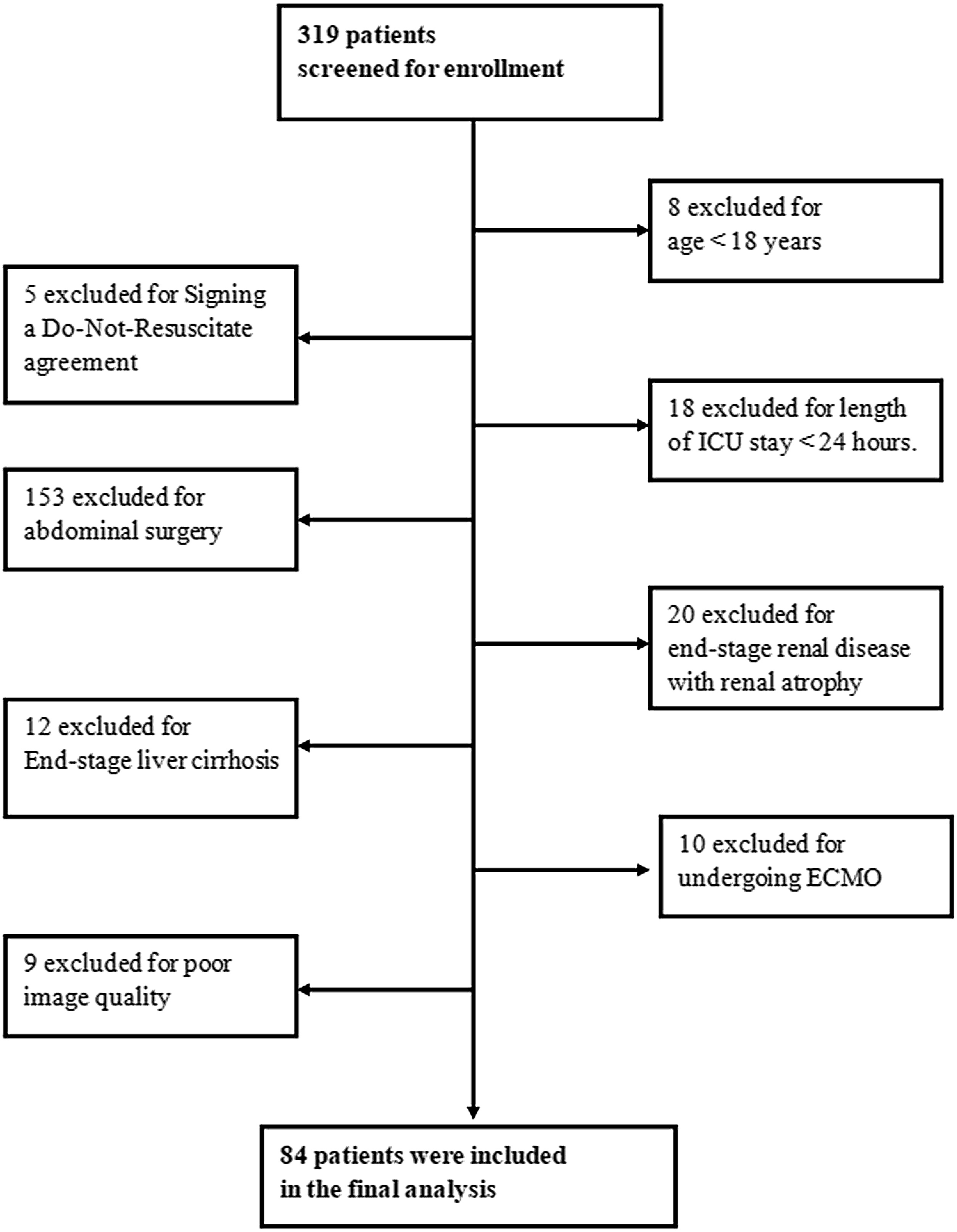
Patient enrollment flowchart for the assessment of venous congestion.

A total of 319 patients were screened for eligibility during the study period. After application of the predefined inclusion and exclusion criteria, 235 patients were excluded, including 9 patients due to inadequate ultrasound image quality. Ultimately, 84 unique patients were included in the final cohort and contributed 116 ultrasound examinations, which were included in the primary analysis. The patient selection process is summarized in Fig. 2.

Baseline characteristics of the cohort are summarized in Table 1. The median age was 65.5 years (interquartile range [IQR] 56.0–75.8), and 63% (n = 53) were male. The median body mass index (BMI) was 22.2 kg/m^2^. Disease severity scores reflected a critically ill population, with a median SOFA score of 7.5 (±3.4) and a median APACHE II score of 18.0 (IQR 13.0–23.0). At the time of ultrasound examination, 49% (57/116) of examinations were performed under invasive mechanical ventilation, and 48% (56/116) were performed while patients were receiving continuous renal replacement therapy (CRRT). In addition, 61% (71/116) of examinations were performed under vasopressor support (e.g., norepinephrine), and atrial fibrillation was present in 6% (7/116) of examinations at the time of assessment. The median ICU length of stay (LOS) was 14.0 days (IQR 6.0–26.8), and the median hospital LOS was 26.5 days (IQR 13.0–39.5). The 28-day mortality rate was 26% (n = 22) (Table S1).

**Table 1:** Patient characteristics and clinical status at the time of examination.

Variable	Overall
Baseline demographics (n = 84)	
Male, n (%)	53 (63)
Age, years	65.5 (56.0, 75.8)
Elderly (≥65 years), n (%)	46 (55)
BMI, kg/m^2^	22.2 ± 3.6
SOFA score	7.5 ± 3.4
Apache II score	18.0 (13.0, 23.0)
Hypertension, n (%)	48 (57)
Diabetes, n (%)	28 (33)
Coronary Heart Disease, n (%)	21 (25)
Chronic Kidney Disease, n (%)	20 (24)
Cirrhosis, n (%)	3 (4)
Sepsis, n (%)	65 (77)
Baseline heart failure (acute/chronic; left/right), n (%)	25(30)
Acute left heart failure, n (%)	17 (20)
Acute right heart failure, n (%)	0 (0)
Chronic left heart failure, n (%)	6 (7)
Chronic right heart failure	2 (2)
Acute Abdominal Disease, n (%)	21 (25)
Exam-time clinical status (n = 116)	
Mechanical Ventilation, n (%)	57 (49)
Vasopressors, n (%)	71 (61)
Inotropes, n (%)	6 (5)
Atrial fibrillation, n (%)	7 (6)
Sinus rhythm, n (%)	109 (94)
CRRT at exam time, n (%)	56 (48)
White Blood Cell, ×10⁹/L	10.8 (7.8, 14.3)
Platelet, ×10⁹/L	171.5 (103.3, 229.3)
Total Bilirubin, μmol/L	17.2 (10.1, 26.0)
ALT	26.8 (13.0, 65.4)
AST	35.1 (25.7, 180.7)
Creatinine, μmol/L	123.0 (80.5, 291.0)
Urea Nitrogen, mmol/L	12.7 (5.6, 18.3)
PaO₂/FiO₂ Ratio, mmHg	293.5 (197.8, 375.8)
Lactate, mmol/L	1.5 (0.9, 3.1)
Urine Output, mL/24h	915.0 (262.5, 1660.0)

Abbreviations: BMI, body mass index; CHD, coronary heart disease; CKD, chronic kidney disease; SOFA, Sequential Organ Failure Assessment; APACHE, Acute Physiology and Chronic Health Evaluation; CRRT, continuous renal replacement therapy; MV, mechanical ventilation; LOS, length of stay. Notes: Data are presented as n (%) for categorical variables and median (interquartile range) for continuous variables. Baseline demographics and comorbidities are shown for the total cohort (N = 84). Clinical status and laboratory data are presented for the total number of examinations (N = 116 examinations).

### Stratification by inferior vena cava diameter and venous congestion

A total of 116 venous Doppler ultrasound examinations were performed across the cohort. Despite a smaller IVC diameter, a considerable proportion of examinations in the <2 cm group demonstrated sonographic evidence of venous congestion. Specifically, venous congestion (Grade 1–3) was identified in 32.8% (21/64) of examinations, including 4.7% (3/64) with Grade 2 and 1.6% (1/64) with Grade 3 congestion. Conversely, 19.2% (10/52) of examinations in the ≥2 cm group showed no Doppler signs of congestion (Fig. S1). Examinations were stratified into two groups according to the conventional IVC diameter cutoff of 2 cm, with detailed characteristics shown in Table S2. The median IVC diameter was 1.6 cm (IQR 1.4–1.8) in the <2 cm group and 2.2 cm (IQR 2.1–2.4) in the ≥2 cm group. Correspondingly, the SCI of IVCwas significantly higher in the ≥2 cm group (0.8, IQR 0.7–0.9) than in the <2 cm group (0.6, IQR 0.4–0.7).

### Correlation between venous congestion grade and ultrasound metrics

**Fig. 3:**
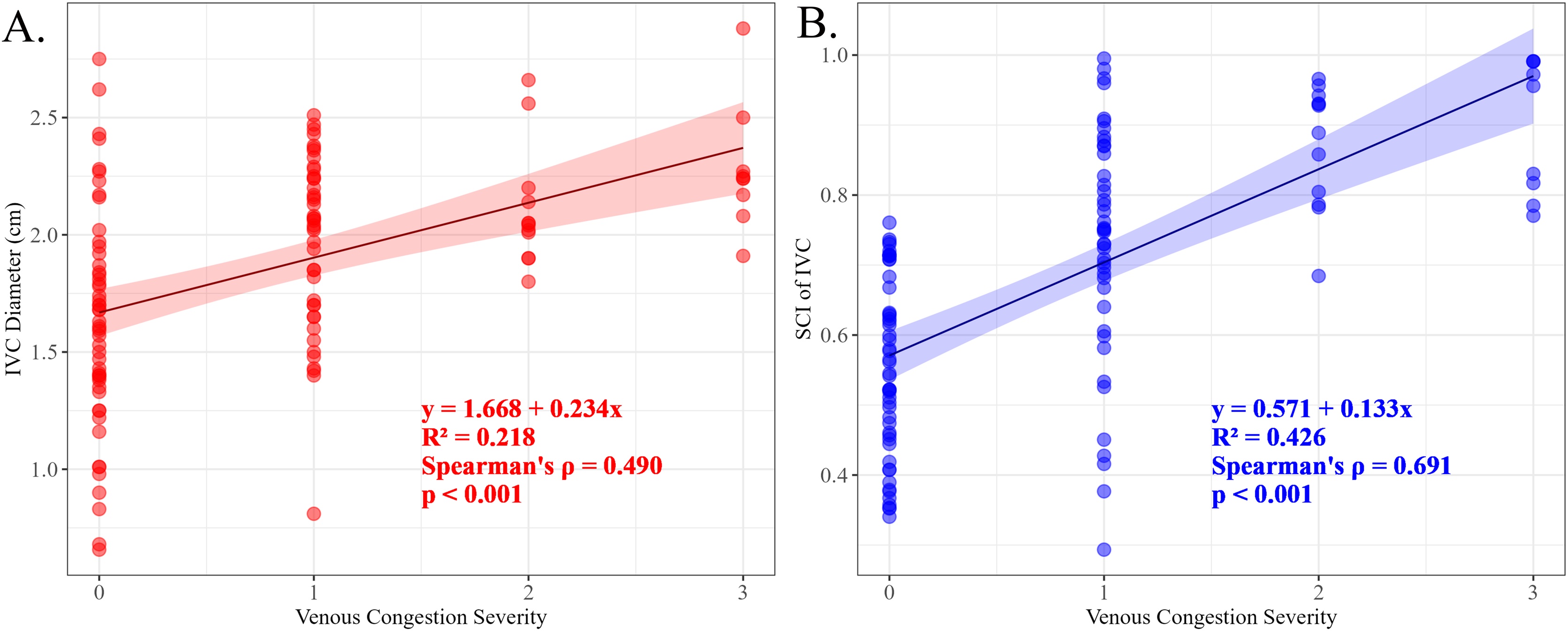
Association between IVC parameters and venous congestion severity in the overall examinations(n = 116). (A) Scatter plot of IVC diameter versus venous congestion severity score. (B) Scatter plot of shape change index (SCI) of IVC versus venous congestion severity score. In both panels, the solid line (red in A, blue in B) represents the linear regression fit, with the corresponding equation and coefficient of determination (R²) annotated. The shaded area around the regression line indicates the 95% confidence interval.

Across all 116 venous Doppler examinations, venous congestion severity was significantly correlated with both ultrasound parameters (Fig. 3). The congestion grade showed a stronger monotonic relationship with the SCI of IVC than with IVC diameter. The IVC diameter demonstrated a moderate positive correlation with congestion grade (Spearman’s ρ = 0.490, p < 0.001), explaining 21.8% of the observed variance (R² = 0.218). The corresponding regression equation was y = 1.668 + 0.234x. In contrast, the SCI of the IVC exhibited a stronger association with venous congestion grade (Spearman’s ρ = 0.691, p < 0.001), accounting for 42.6% of the variance (R² = 0.426). The fitted regression equation was y = 0.571 + 0.133x. These findings indicate that SCI provides a more consistent reflection of the hemodynamic changes associated with venous congestion.

### Predictive performance for venous congestion

**Fig. 4:**
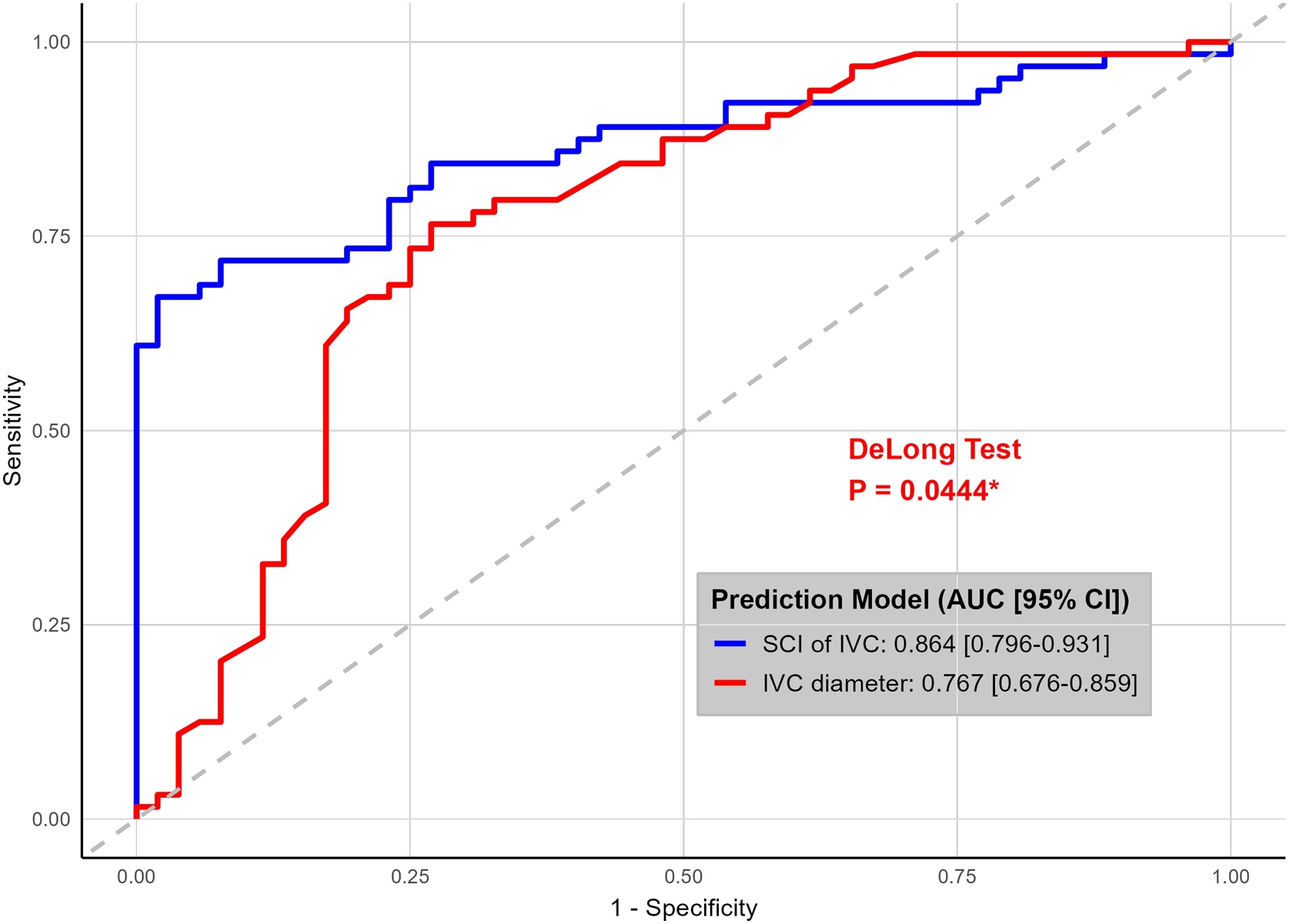
Receiver operating characteristic (ROC) curves for discriminating examinations with venous congestion. The models based on the SCI of IVC (blue line) and IVC diameter (red line) are evaluated forpredicting venous congestion (VExUS grade ≥1 vs grade 0). The area under the curve (AUC) with 95% confidence interval is shown for each model. The P-value from the DeLong test for comparing the two ROC curves in each panel is annotated, with significance levels indicated as * (*P* < 0.05).

ROC analysis demonstrated that the SCI of IVC outperformed IVC diameter in discriminating examinations with venous congestion when using a unified and clinically applicable endpoint (VExUS grade ≥1 vs grade 0). The SCI achieved an area under the curve (AUC) of 0.864 (95% CI 0.796–0.931), which was significantly higher than the AUC of 0.767 (95% CI 0.676–0.859) for IVC diameter (p = 0.044, DeLong test) (Fig. 4, Table 2). The optimal cutoff value for SCI was 0.742, yielding a sensitivity of 67.2% and a specificity of 98.1%. To further characterize the diagnostic behavior of SCI and IVC diameter across different stages of venous congestion, severity-stratified ROC analyses were performed for mild (Grade ≥1), moderate (Grade ≥2), and severe (Grade ≥3) congestion. In addition, reclassification analyses and decision curve analyses were conducted as exploratory assessments of model performance. Exploratory severity-stratified analyses suggested that the diagnostic advantage of SCI over IVC diameter varied across congestion stages, with a trend toward greater separation in moderate congestion. These secondary and exploratory analyses are presented in the Supplementary Materials (Fig S2; Tables S3–S4).

**Table 2:** Diagnostic performance of SCI of IVC and IVC diameter for predicting grade 1 of venous congestion.

Severity Grade	Metric	AUC 95% CI	Cut-off	Sensitivity	Specificity	P-value
Grade 1	SCI of IVC	0.864(0.796−0.931)	0.742	0.672	0.981	0.044
Grade 1	IVC Diameter	0.767(0.676−0.859)	1.84	0.766	0.731	

Abbreviations: IVC, inferior vena cava; SCI, shape change index; AUC, area under the receiver operating characteristic curve; CI, confidence interval. Note: Patients were categorized into two groups based on clinical criteria: Any venous congestion​ (Grade 1 or higher) and No congestion. The P-values in the last column represent the statistical significance of the difference in AUC between the SCI of IVC model and the IVC Diameter model for each severity grade, as determined by the DeLong test.

### Parameter discrepancies stratified by IVC diameter and SCI cutoffs

Based on the established diagnostic thresholds for venous congestion assessment, patients were stratified into groups according to the IVC diameter (≥2.0 cm) and the SCI of IVC using the optimal cut-off (0.742) for predicting mild congestion, as determined by prior ROC analysis. As shown in Table 3, no significant differences were observed between groups regarding age, sex, or comorbidities. However, in terms of disease severity, patients in the SCI ≥ 0.742 group had a significantly higher SOFA score than those in the SCI < 0.742 group (8.5 [6.0, 11.0] vs. 7.0 [5.0, 9.0], *p* = 0.015). Analysis of clinical status at the time of examination (Table 3) revealed no significant differences in the proportions of patients requiring mechanical ventilation, vasopressors, or inotropes between the different subgroups. Notably, a higher proportion of patients with an IVC diameter ≥2 cm had atrial fibrillation at examination compared to those with a diameter <2 cm (14% vs. 0%, *p* = 0.003). Furthermore, the 24-hour urine output was significantly lower in the SCI ≥ 0.742 group than in the SCI < 0.742 group (1050 [183, 1938] mL vs. 1600 [789, 2683] mL, *p* = 0.039).

**Table 3:** Baseline characteristics and exam-time clinical status stratified by IVC diameter and SCI of IVC thresholds.

	IVC diameter <2 (n = 42)	IVC diameter ≥2 (n = 42)	*P*	SCI of IVC ＜0.742 (n = 48)	SCI of IVC ≥ 0.742 (n = 36)	*P*
Baseline characteristics (n = 84)						
Male, n (%)	28 (67)	25 (60)	0.651	32 (67)	21 (58)	0.497
Age, years	65.0 (51.8, 75.0)	67.0 (59. 8,76.0)	0.376	66.0 (56.0, 75.8)	65.0 (51.0, 75.5)	0.820
Hypertension, n (%)	21 (50)	27 (64)	0.27	30 (63)	18 (50)	0.274
Diabetes, n (%)	15 (36)	13 (31)	0.817	13 (27)	15 (42)	0.242
CHD, n (%)	11 (26)	10 (24)	1.0	10 (21)	11 (31)	0.446
CKD, n (%)	9 (21)	11 (26)	0.798	10 (28)	10 (28)	0.605
Hepatic Cirrhosis, n (%)	0 (0)	3 (7)	0.241	0 (0)	3 (8)	0.075
Sepsis, n (%)	33 (79)	32 (76)	1.0	39 (81)	26 (72)	0.431
Heart failure, n (%)	15 (36)	10 (24)	0.340	12 (25)	13 (36)	0.337
Acute Abdominal Disease, n (%)	9 (21)	12 (29)	0.615	13 (27)	8 (22)	0.800
SOFA score	7.0 (4.8, 9.0)	8.0 (5.8, 11.0)	0.053	7.0 (5.0, 9.0)	8.5(6.0, 11.0)	0.015
Apache II score	17.0 (12.8, 19.0)	19.0 (13.0, 24.0)	0.155	17.0 (13.0, 19.0)	19.5(13.0, 23.0)	0.118
Exam-time clinical status (n = 116)						

	n = 64	n = 52		n = 72	n = 44	
MV, n (%)	38 (60)	32 (62)	0.850	42 (58)	28 (64)	0.696
Vasopressors, n (%)	35 (55)	34 (65)	0.260	39 (54)	30 (68)	0.173
Inotropes, n (%)	2 (3)	1 (2)	1.000	0 (0)	3 (7)	0.052
Atrial fibrillation, n (%)	0 (0)	7 (14)	0.003	1 (1)	6 (14)	0.012
Sinus rhythm, n (%)	62 (97)	45 (87)	0.076	69 (99)	38 (86)	0.081
CRRT, n (%)	30 (47)	26 (50)	0.852	30 (42)	26 (59)	0.086
WBC, ×10⁹/L	9.6 (6.3, 13.2)	9.9 (6.0, 15.8)	0.378	9.6 (6.4, 12.1)	10.9 (5.6, 16.2)	0.273
Platelet, ×10⁹/L	165.0 (98.8, 241.3)	143.0 (77.3, 187.0)	0.147	168.0 (98.8, 221.5)	135.0 (75.0, 187.0)	0.114
Tbil, μmol/L	17.7 (8.3, 33.0)	19.4 (10.7, 40.8)	0.291	15.9 (8.3, 28.8)	23.9 (12.6, 40.6)	0.033
ALT, μmol/L	27.2 (14.0, 55.7)	29.7 (12.9, 59.9)	0.716	27.7 (13.3, 52.2)	27.2 (13.0, 73.0)	0.922
AST, μmol/L	39.3 (19.7, 100.3)	40.5 (26.1, 108.2)	0.494	40.1 (20.9, 90.3)	40.5(23.6, 110.4)	0.697
Creatinine, μmol/L	110.0 (69.5, 225.0)	107.5 (78.5, 228.5)	0.627	105.0 (69.5, 214.0)	136.0 (82.8, 272.5)	0.115
BUN, mmol/L	9.3 (4.4, 18.0)	11.0 (6.3, 18.2)	0.297	9.3 (4.4, 16.5)	11.2 (6.0, 20.0)	0.099
Urine, mL/24h	1630 (300, 2618)	1260 (335, 2033)	0.326	1600 (789, 2683)	1050 (183, 1938)	0.039
Lactate, mmol/L	1.5 (0.9, 2.2)	1.4 (1.0, 2.5)	0.657	1.4 (0.9, 2.2)	1.5 (0.9, 2.9)	0.597
PaO₂/FiO₂, mmHg	301 (216, 386)	278 (188, 342)	0.175	292 (214, 384)	297 (188, 348)	0.599

Abbreviations: IVC, inferior vena cava; SCI, Shape Change Index; CHD, coronary heart disease; CKD, chronic kidney disease; SOFA, Sequential Organ Failure Assessment; APACHE II, Acute Physiology and Chronic Health Evaluation II; MV, mechanical ventilation; CRRT, continuous renal replacement therapy; WBC, white blood cell; Tbil, total bilirubin; ALT, alanine aminotransferase; AST, aspartate aminotransferase; BUN, blood urea nitrogen; PaO₂/FiO₂, arterial oxygen partial pressure to fractional inspired oxygen ratio. Notes: Data are presented as n (%) for categorical variables and median (interquartile range) for continuous variables. Patients were stratified into two groups based on the established cut-off values for venous congestion: IVC diameter ≥2.0 cm and SCI of IVC ≥ 0.742. Baseline demographics and comorbidities are shown for the total cohort (N = 84). Clinical status and laboratory data are presented for the total number of examinations (N = 116 examinations). P-values were derived from the chi-square test or Fisher’s exact test for categorical variables and the Mann–Whitney U test for continuous variables, as appropriate.

## Discussion

The present study challenges the conventional VExUS scoring framework, which defines an IVC diameter ≤2 cm as Grade 0 and uses this threshold to exclude venous congestion [5]. Our findings revealed that 32.8% of examinations with an IVC diameter <2 cm still exhibited venous congestion (Grades 1–3), including 4.7% with moderate and 1.6% with severe congestion. This observation highlights a fundamental limitation in relying solely on absolute IVC diameter as a surrogate marker for venous congestion. Consistent with this limitation, our unified ROC analysis using a single clinically applicable endpoint (any venous congestion, VExUS grade ≥1 vs grade 0) demonstrated that SCI provided significantly better discrimination than IVC diameter (AUC 0.864 vs 0.767, DeLong p = 0.044), with optimal cutoffs of 0.742 for SCI and 1.84 cm for IVC diameter. Beyond its statistical performance, SCI-based stratification also demonstrated meaningful clinical coherence. Examinations with an SCI ≥ 0.742 were associated with higher illness severity as reflected by higher SOFA scores, higher total bilirubin levels, a greater prevalence of atrial fibrillation, and lower urine output, all of which are well-recognized manifestations of venous congestion.

This finding highlights a key limitation of relying solely on absolute IVC diameter thresholds to assess venous congestion, as fixed cutoffs may fail to capture earlier or evolving stages of elevated venous pressure. In this context, the SCI of IVC provides incremental diagnostic value by incorporating IVC morphological deformation rather than diameter alone. Even when the absolute IVC size remains within conventionally “normal” limits, increased venous pressure may alter the relative geometry of the vessel, resulting in detectable shape changes. By capturing these pressure-related morphological features, SCI appears better suited to identify venous congestion that would otherwise be overlooked using diameter-based criteria alone. These observations support the potential role of SCI as a complementary refinement to the existing VExUS framework, particularly at the initial IVC assessment step, where reliance on a single diameter cutoff may underestimate the burden of venous congestion in critically ill patients.

Several factors may explain this discrepancy. First, substantial interindividual variability in IVC diameter exists and correlates strongly with anthropometric parameters such as height, weight, and body surface area [17,18]. Asian populations, who generally have smaller body habitus, exhibit correspondingly smaller baseline IVC diameters [19]. Thus, applying a universal cutoff value derived from Western populations may introduce systematic bias when assessing non-Western cohorts. Second, inherent technical limitations in ultrasound imaging contribute to measurement variability. Minor probe misalignment in the longitudinal view can shift the imaging plane away from the vessel’s centerline, resulting in the so-called “cylinder effect” and underestimation of the true diameter [[20], [21], [22]]. This error is amplified when the IVC is laterally displaced. Notably, a median medial displacement of 4 mm can produce a diameter error of up to 17% on two-dimensional ultrasound [20]. Conversely, in patients with IVC diameter >2 cm but no congestion, elliptical or irregular IVC morphology under low CVPmay lead to overestimation of the diameter, as the long-axis view may capture the maximal dimension rather than the true cross-sectional average [15,[20], [21], [22]].

Given these methodological and physiological constraints, the SCI of IVC offers a more robust alternative. In this study, the SCI of IVC demonstrated consistently stronger associations with venous congestion severity than IVC diameter, showing higher correlation coefficients and explaining nearly twice the variance. Its superior diagnostic performance across congestion grades, supported by reclassification analyses, suggests that SCI captures venous pressure–related morphological changes beyond static diameter measurements.

Our findings are consistent with previous investigations demonstrating that the SCI of IVC correlates more closely with CVP than absolute IVC diameter [23]. In this context, our exploratory analyses suggest that SCI may offer additional value in identifying moderate venous congestion. This observation is clinically relevant, as organ dysfunction at this stage is often functional and potentially reversible [24,25]. From a pathophysiological perspective, moderate congestion represents a transitional state in which venous pressure is sufficiently elevated to induce morphological deformation of the IVC, while not yet reaching the physiological ceiling observed in advanced congestion. Accordingly, SCI may be particularly sensitive to these intermediate morphological changes. In contrast, in cases of severe venous congestion, the diagnostic advantage of SCI appeared to diminish, with the performance of SCI and IVC diameter converging toward a physiological ceiling. This observation is consistent with prior findings indicating that SCI loses sensitivity once CVP exceeds a high threshold [23]. Under conditions of extreme venous distension, the IVC assumes a near-circular configuration, resulting in limited residual morphological variability and a reduced capacity for SCI to provide additional discriminatory information.

It is important to note that the VExUS score fundamentally reflects the pressure gradient between mean systemic filling pressure and CVP, which governs venous return [24]. While SCI captures structural deformation caused by elevated venous pressure, it cannot distinguish among the underlying causes of congestion—such as volume overload, right ventricular dysfunction, or decreased venous compliance, etc. Therefore, SCI should be interpreted in conjunction with global hemodynamic and perfusion parameters to achieve a comprehensive assessment of volume status.

This study has several limitations. First, as a single-center observational study, the sample size was modest, which may limit generalizability and the external validity of the proposed SCI cutoff values. Second, CVP measurements were only available in a subset of cases, and thus were not used as a reference standard in the primary analyses, which focused on noninvasive ultrasound indices for venous congestion assessment. Third, right ventricular (RV) size and function were not systematically evaluated in this study. While RV parameters could offer additional pathophysiological insights, the study primarily assessed IVC-based metrics—particularly the SCI of IVC—within the VExUS framework. Further research is needed to explore the interactions between RV function, venous congestion, and ultrasound-derived metrics. Finally, this study focused primarily on diagnostic correlation rather than prognostic outcomes. Future investigations should explore associations between SCI-guided assessments and clinically relevant endpoints such as acute kidney injury incidence and mortality.

## Conclusion

In summary, this study demonstrates that the SCI of IVC provides superior diagnostic performance compared with conventional IVC diameter measurements for identifying venous congestion in critically ill patients, with particular relevance in the detection of less advanced congestion. Incorporation of SCI as a complementary measure may improve recognition of venous congestion when reliance on absolute IVC diameter alone is insufficient, within the context of bedside ultrasound assessment. Future multicenter prospective studies are needed to validate the optimal diagnostic threshold of SCI and to further define its role within the existing VExUS framework.

## CRediT authorship contribution statement

Lizhi Li: Data collection, Data curation, Data management, Statistical analysis, Visualization, Data validation, Manuscript writing. Yuehua Xu: Data collection, Data curation, Draft manuscript preparation. Xiukai Chen: Manuscript review. Wei Huang: Conceptualization, Resource coordination, Data collection, Supervision, Manuscript review and revision. All authors have read and approved the final version of the manuscript.

## Consent for publication

Not applicable.

## Ethical approval and consent to participate

The study protocol was approved by the Hospital Ethics Committee (Approval No. 098, 2025). The requirement for informed consent was waived as all data were collected as part of standard medical care and anonymized prior to analysis.

## Funding

This work was supported by the Chinese National Natural Science Foundation (No.82402515)

## Availability of data and materials

The datasets used and/or analysed during the current study are available from the corresponding author on reasonable request.

## Declaration of competing interest

The authors declare no competing interests.

## Figures and Tables

**Figure:**
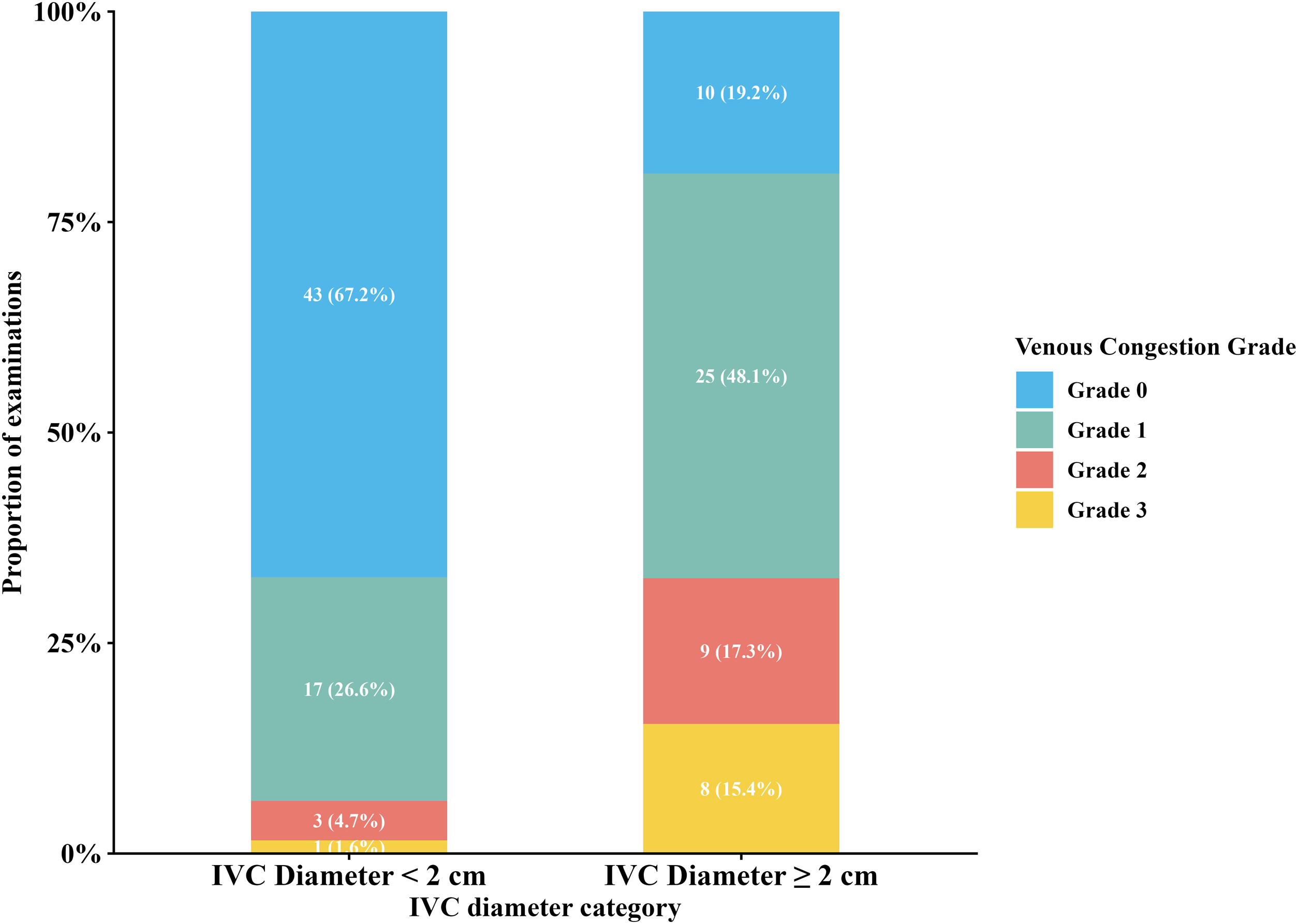


**Figure:**