# Supplementary material for: Assessing venous congestion in critical illness: advantages of the inferior vena cava shape change index over diameter
Source: Ann Intensive Care. 2026 Feb 9;16:100032. doi: 10.1016/j.aicoj.2026.100032 (PMC12934418; doi:10.1016/j.aicoj.2026.100032)
Supplement: Supplementary file 1 [file mmc1.docx]

**Table S1. Clinical Outcomes of the study population**

| **Variable​​** | **​​Overall** |
| --- | --- |
| MV duration, h | 108.0（0.0, 290.0] |
| Hospital LOS, d | 26.5（13.0, 39.5] |
| ICU LOS, d | 14.0（6.0, 26.8] |
| 28-day Mortality, n (%) | 22 (26) |

Abbreviations: MV, mechanical ventilation; LOS, length of stay.
