# Supplementary material for: Assessing venous congestion in critical illness: advantages of the inferior vena cava shape change index over diameter
Source: Ann Intensive Care. 2026 Feb 9;16:100032. doi: 10.1016/j.aicoj.2026.100032 (PMC12934418; doi:10.1016/j.aicoj.2026.100032)
Supplement: Supplementary file 2 [file mmc2.docx]

**Table S2. Distribution of venous congestion parameters stratified by inferior vena cava (IVC) diameter groups​**

| Variable | Overall^1^ (n=116) | Diameter < 2 cm (n=64) | Diameter ≥ 2 cm (n=52) |
| --- | --- | --- | --- |
| **​​IVC diameter, cm​​** | 1.9 [1.6, 2.2] | 1.6 [1.4, 1.8] | 2.2 [2.1, 2.4] |
| **​​SCI of IVC​​** | 0.7 [0.5, 0.8] | 0.6 [0.4, 0.7] | 0.8 [0.7, 0.9] |
| **​​IVC Short Diameter, cm​​** | 1.6 ± 0.5 | 1.3 ± 0.5 | 2.0 ± 0.3 |
| **​​IVC Long Diameter, cm​​** | 2.3 [2.0, 2.7] | 2.3 [1.8, 2.5] | 2.6 [2.2, 2.7] |
| **Hepatic vein status​**​, n(%) |  |  |  |
| Normal | 73 (62.9) | 50 (78.1) | 23 (44.2) |
| Mildly abnormal | 31 (26.7) | 11 (17.2) | 20 (38.5) |
| Severely abnormal | 12 (10.3) | 3 (4.7) | 9 (17.3) |
| Unable to assess | 0 (0) | 0 (0) | 0 (0) |
| **Portal vein status​**​, n(%) |  |  |  |
| Normal | 65 (56.0) | 49 (76.6) | 16 (30.8) |
| Mildly abnormal | 35 (30.2) | 13 (20.3) | 22 (42.3) |
| Severely abnormal | 15 (13.0) | 2 (3.1) | 13 (25.0) |
| Unable to assess | 1 (0.9) | 0 (0) | 1 (1.9) |
| **Renal vasculature status​**​, n(%) |  |  |  |
| Normal | 80 (69.0) | 46 (71.9) | 34 (65.4) |
| Mildly abnormal | 14 (12.1) | 7 (11.0) | 7 (13.5) |
| Severely abnormal | 8 (6.9) | 0 (0) | 8 (15.4) |
| Unable to assess | 14 (12.1) | 11 (17.2) | 3 (5.8) |
| **VExUSGrade​**​, n(%) |  |  |  |
| 0 | 64 (55.2) | 64 (100.0) | 0 (0) |
| 1 | 35 (30.2) | 0 (0) | 35 (67.3) |
| 2 | 9 (7.8) | 0 (0) | 9 (17.3) |
| 3 | 8 (6.9) | 0 (0) | 8 (15.4) |
| ​**​Venous Congestion Grade​**​, n(%) |  |  |  |
| 0 | 52 (44.8) | 42 (65.6) | 10 (19.2) |
| 1 | 43 (37.1) | 18 (28.1) | 25 (48.1) |
| 2 | 12 (10.3) | 3 (4.7) | 9 (17.3) |
| 3 | 9 (7.8) | 1 (1.6) | 8 (15.4) |

Abbreviations: IVC, inferior vena cava; SCI, Sonographic Congestion Index; VExUS, Venous Excess Ultrasound Score.

^1^The total number of ultrasound examinations was 116, performed on 84 patients. Percentages for categorical variables are calculated based on the total number of examinations within each diameter group.
