# Supplementary material for: Assessing venous congestion in critical illness: advantages of the inferior vena cava shape change index over diameter
Source: Ann Intensive Care. 2026 Feb 9;16:100032. doi: 10.1016/j.aicoj.2026.100032 (PMC12934418; doi:10.1016/j.aicoj.2026.100032)
Supplement: Supplementary file 3 [file mmc3.docx]

**Table S3. Diagnostic performance of SCI of IVC and IVC diameter for predicting different grades of venous congestion.​**

| Severity Grade | Model | AUC | 95% CI | Optimal Cut-off | Sensitivity | Specificity | P-value |
| --- | --- | --- | --- | --- | --- | --- | --- |
| Mild | SCI of IVC | 0.864 | 0.796-0.931 | 0.742 | 0.672 | 0.981 | 0.044 |
| Mild | IVC Diameter | 0.767 | 0.676-0.859 | 1.84 | 0.766 | 0.731 |  |
| Moderate | SCI of IVC | 0.899 | 0.839-0.959 | 0.766 | 0.952 | 0.811 | 0.004 |
| Moderate | IVC Diameter | 0.75 | 0.658-0.841 | 1.88 | 0.952 | 0.579 |  |
| Severe | SCI of IVC | 0.888 | 0.805-0.971 | 0.766 | 1 | 0.729 | 0.302 |
| Severe | IVC Diameter | 0.806 | 0.702-0.910 | 2.08 | 0.889 | 0.701 |  |

**Abbreviations:**  IVC, inferior vena cava; SCI, shape change index; AUC, area under the receiver operating characteristic curve; CI, confidence interval. **Note:​**​ The P-values in the last column represent the statistical significance of the difference in AUC between the SCI of IVC model and the IVC Diameter model for each severity grade, as determined by the DeLong test.
