# Supplementary material for: Assessing venous congestion in critical illness: advantages of the inferior vena cava shape change index over diameter
Source: Ann Intensive Care. 2026 Feb 9;16:100032. doi: 10.1016/j.aicoj.2026.100032 (PMC12934418; doi:10.1016/j.aicoj.2026.100032)
Supplement: Supplementary file 4 [file mmc4.docx]

**Table S4. Improvement in predictive performance for venous congestion: SCI of IVC vs. IVC diameter (Bootstrap Analysis of NRI and IDI)​**

| Severity  Grade | Metric | Initial Estimate (SE) | Initial 95% CI | Bootstrap Estimate (R=1000) | Bootstrap 95% CI | Bootstrap P-value |
| --- | --- | --- | --- | --- | --- | --- |
| ​​Mild​​ | ​​NRI | 0.1274 (0.1014) | -0.0677 to 0.3226 | 0.7091 | -0.0446 to 1.2425 | 0.086 |
| ​​Mild​​ | ​​IDI | - | - | 0.165 | -0.0191 to 0.3439 | 0.088 |
| ​​Moderate​​ | ​​NRI | 0.3865 (0.1483) | 0.0959 to 0.6624 | 0.6015 | 0.0152 to 1.0996 | ​​0.012​​ |
| ​​Moderate​​ | ​​IDI | - | - | 0.2362 | 0.0593 to 0.4532 | ​​0.006​​ |
| ​​Severe​​ | ​​NRI | -0.1111 (0.1143) | -0.3693 to 0.1471 | 0.2046 | -0.5811 to 0.8323 | 0.398 |
| ​​Severe​​ | ​​IDI | - | - | 0.0921 | -0.1628 to 0.3874 | 0.402 |

**Abbreviations:​**​ IVC, inferior vena cava; SCI, Sonographic Congestion Index; NRI, Net Reclassification Improvement; IDI, Integrated Discrimination Improvement; SE, standard error; CI, confidence interval.​**​Notes:​**​ Data are presented as estimate (SE) and 95% CI. The initial estimates and CIs for NRI were calculated using standard asymptotic methods. Bootstrap estimates, CIs, and P-values were derived from 1000 bootstrap samples to assess the stability and significance of the improvement. The bootstrap P-value represents the probability of observing an improvement statistic ≤ 0 under the null hypothesis of no improvement. A dash (-) indicates that the metric is not applicable or was not calculated using asymptotic methods.
